# Cracking the Code of Human Diseases Using Next-Generation Sequencing: Applications, Challenges, and Perspectives

**DOI:** 10.1155/2015/161648

**Published:** 2015-11-19

**Authors:** Vincenza Precone, Valentina Del Monaco, Maria Valeria Esposito, Fatima Domenica Elisa De Palma, Anna Ruocco, Francesco Salvatore, Valeria D'Argenio

**Affiliations:** ^1^CEINGE-Biotecnologie Avanzate, Via G Salvatore 486, 80145 Naples, Italy; ^2^Department of Molecular Medicine and Medical Biotechnologies, University of Naples Federico II, Ed. 19, Via Sergio Pansini 5, 80131 Naples, Italy; ^3^IRCCS-Fondazione SDN, 80143 Naples, Italy

## Abstract

Next-generation sequencing (NGS) technologies have greatly impacted on every field of molecular research mainly because they reduce costs and increase throughput of DNA sequencing. These features, together with the technology's flexibility, have opened the way to a variety of applications including the study of the molecular basis of human diseases. Several analytical approaches have been developed to selectively enrich regions of interest from the whole genome in order to identify germinal and/or somatic sequence variants and to study DNA methylation. These approaches are now widely used in research, and they are already being used in routine molecular diagnostics. However, some issues are still controversial, namely, standardization of methods, data analysis and storage, and ethical aspects. Besides providing an overview of the NGS-based approaches most frequently used to study the molecular basis of human diseases at DNA level, we discuss the principal challenges and applications of NGS in the field of human genomics.

## 1. Introduction

DNA sequencing is the process of determining the exact order of the nucleotides in a DNA segment, corresponding to single gene(s) or to a variety of molecules in the case of the whole genome, or a large part of it. Therefore, techniques able to do this have radically changed the course of molecular research in all its fields of application. In the last 30 years, the so-called Sanger sequencing has been the most widely used sequencing technology worldwide [1]. Its use peaked with the human genome project, which, in 2001, elucidated the entire human genome [2, 3]. Although Sanger sequencing is now completely automated, it is a method based on one or more amplicons that sequence relatively small strings of DNA rather than a complete gene or a set of genes in the human genome. Consequently, it is an expensive and time-consuming procedure if used to determine the entire chromosomal asset of a single organ tissue, or even cell.

In the last ten years, novel technologies, collectively called “next-generation sequencing” (NGS), have become available and have dramatically increased the throughput of DNA sequencing, thereby simultaneously reducing its costs [4]. Just to give an idea of what this means, it took more than 10 years to elucidate the first human genome sequence and it cost USD 3 billion. Using NGS instruments, the entire genome sequence of an individual can now be elucidated in less than 1 year and at a much lower cost [5]. It is expected that the sequencing of the entire genome of an individual will soon cost a few thousand USD (now 2,000 USD/genome without interpretation) [6].

These advances in genomic technologies have accelerated the search for genetic causes of human diseases and have enabled investigators to answer previously unapproachable questions about disease pathogenesis. In recent years, several NGS-based approaches have been devised and validated to facilitate the study of the molecular basis of human diseases [6]. The ultimate aims are (i) to develop novel, sensitive, accurate, and cost- and time-effective pipelines for molecular diagnostics and (ii) to elucidate the mechanisms involved in disease development and so to identify novel diagnostic, prognostic, and therapeutic markers [7]. In fact, NGS technologies can be easily applied to a wide range of research fields; thus far, they have been successfully used to analyze target regions of the human genome ranging in size from the entire exome to a restricted number of genes or a single amplicon [8–10]. In addition to the detection of nucleotide variants on DNA regions, NGS-based strategies can shed light on the DNA methylation status, at both single gene and genome-wide level [11]. Importantly, apart from the human genome, NGS gave immeasurable impetus to metagenomics. In fact, work based on NGS revealed the complexity of the microbiota not only in diverse tissues and organs, but also in relation to a variety of physiological conditions (e.g., sex, age, and circadian rhythm) as well as in pathological conditions [12].

Novel NGS-based strategies are continuously being developed and it is conceivable that these technologies will become even more routine also for diagnostic purposes, particularly in view of the progressive simplification of NGS protocols, the reduction in the operator's “hands on” work, and the launch of the “benchtop” NGS platforms. Notably, the integration of data obtained using several NGS-based strategies could shed light on the mechanisms involved in disease development and, in turn, reveal targets that can be manipulated to obtain better identification, stratification, and treatment of patients.

Here, we review the main NGS-based approaches currently used to study the molecular basis of human diseases at DNA level and discuss the main advantages, principal applications, and possible limitations of each.

## 2. NGS-Based Analytic Approaches for the Study of Human Diseases

### 2.1. Identification of DNA Sequence Variants

The study of entire genomes is faster and cheaper with NGS techniques than with conventional Sanger sequencing [13, 14]. However, the entire sequencing of a large number of samples is not yet feasible for routine use due to the cost, time, and infrastructures required. Consequently, various approaches to specifically enrich target genomic regions simultaneously thereby allowing barcoding of samples for sequence multiplexing have been developed and can be divided into polymerase chain reaction- (PCR-) based and non-PCR-based strategies. Both strategies can be used for NGS library preparation; the choice of the most appropriate strategy depends on the size of the target regions, the number of samples to be analyzed, the cost and time required, and the biological questions to be addressed, as reviewed in the following subsections. The specific analytic features and applications to human diseases of each of these approaches are summarized in Tables 1 and 2, respectively.

#### 2.1.1. PCR-Based Strategies

PCR-based strategies have been the most widely used presequencing strategies since they are perfectly compatible with Sanger sequencing and also with all NGS methodologies and instruments: the amplicons resulting from amplification have NGS-platform-specific adapters ligated to their ends. These represent a library that is suitable for downstream sequencing reactions [8]. Since barcode sequence tags can be added during this step, sample multiplexing is possible [15]. Amplicon sequencing refers to ultradeep sequencing of PCR products obtained from one or several specific targets in order to determine, for example, genetic variations contained in precise portions of the genome. However, PCR amplification is too laborious for large-scale NGS downstream applications and risks being a bottleneck in the sample preparation workflow; consequently, more appropriate PCR procedures were developed. Below we briefly review long-range PCR, multiplex PCR, and microdroplet PCR [16–18].

*Long-Range PCR. *Long-range PCR procedures can be used to analyze up to several hundreds of kilobases (kb), such as the entire sequence of a single gene of interest. This enrichment strategy requires the design of large overlapping amplicons ranging in size from 2 to 12 kb. Each amplicon must be individually amplified, purified, and quantified. Then, all amplicons from the same sample can be pooled in equimolar amounts and used to obtain a library obtained using shotgun protocols. Since barcoded adapters can be added to the mixture in this step, sample multiplexing is allowed.

Long-range PCR has been used to obtain a comprehensive genetic map of a specific genomic locus, 136 kb on chr8q24, which is related to an increased risk of prostate and colon cancer [16]. The same strategy has been successfully used for the complete sequence analysis of large genes such as dystrophin, BRCA1, and BRCA2 [19, 20]. Although long-range PCR can be used to analyze an entire gene of interest including introns, the promoter, and the 3′ untranslated regions (UTR), it has some limitations. Long PCRs require high fidelity Taq polymerase, and amplifications conditions could require a long optimization time. Therefore, this approach is not suitable for large-scale applications and should be limited to the analysis of single genes. 

*Multiplex PCR. *Multiplex PCR is a PCR improvement that enables the simultaneous analysis of multiple targets. In this case, amplicons of different sizes are amplified in one or more multiplex mixtures. Next, a nested PCR is carried out for adapter ligation and amplicon barcoding; all reactions from the same sample are pooled to obtain a multiple amplicon library/sample so that several tagged libraries can be sequenced together.

Multiplex PCR is especially suitable for the simultaneous sequencing of 15 and even more exons, in one or more genes of interest, which results in significant savings in terms of time and costs. Indeed, by targeting multiple genes simultaneously, more comprehensive information can be obtained from a single test run. Notwithstanding these advantages, multiplex PCR can be used only for the analysis of specific known, not very large, carefully selected DNA regions, and the efficiency of the procedure depends strictly on primer design. Hence, time and costs increase in proportion to the number of genes analyzed and their size.

Various commercial kits are available for multiplex PCR-based enrichment. This approach has been used to study the molecular basis of several inherited diseases, namely, familial hypercholesterolemia [17], Alport syndrome [21], hematologic malignancies [22, 23], and cystic fibrosis [24, 25]. It has been also used to identify both somatic and germline mutations in cancer-related genes [26, 27]. Hansen et al., for example, developed an amplicon-based sequencing workflow for the analysis of four genes, predisposing to colorectal cancer [28]. This approach has also been used for the molecular analysis of the BRCA genes [29–33]. Finally, Sie et al. successfully used a multiplex PCR approach for somatic mutation profiling [34].

Taken together, these data indicate that multiplex PCR-based approaches are reliable in detecting mutations in small targets and suggest they could be used in routine diagnostic workflows thanks to their accuracy, speed, and cost-saving features. One advantage of multiplex PCR-based methods is the homogeneous distribution of amplicons, which in turn results in a homogeneous distribution of sequencing reads and a more uniform coverage. This is particularly important when these methods are used in clinical diagnostic procedures. In fact, in such cases, the entire target region must be sequenced without leaving any small sequence gaps, which might harbor the disease-causing mutation. In this context, it is advisable to verify the presence and relative abundance of the different amplicons using presequencing fragment analysis protocols. This verification step not only ensures a high sequence quality and a target coverage close to 100% but also may detect deleterious mutations [35].

*Microdroplet PCR. *Microdroplet PCR is a target enrichment procedure based on high throughput multiplex amplification. Each amplicon is amplified within a droplet of a water-in-oil emulsion to prevent cross-contamination in the reaction tube. Thus, while classical PCR is the amplification of one amplicon in one reaction tube and multiplex PCR is the amplification of a mixture of amplicons in the same reaction tube, microdroplet PCR is the amplification of up to 20,000 different amplicons each inside its own droplet all of which are collected in the same reaction tube. Droplets are generated by fully automated systems that use microfluidic supports (e.g., the RainDance ThunderStorm System). Primer droplets, each containing just one primer pair, are produced to specifically amplify each target of interest. In the same way, each DNA molecule is eluted in a droplet together with the amplification reaction mixture. Each of these two kinds of droplets flows along their microfluidic channel to the merge area, where they fuse into a single droplet that contains one primer pair and one DNA molecule. All the droplets are collected in a reaction tube and amplified before sequencing. This approach can be used to enrich targets within the human genome [18].

Microdroplet PCR has been applied in cancer [36] and in inherited diseases such as congenital muscular dystrophy [37]. In addition, it has been proposed for large-scale, targeted bisulfite sequencing for methylation profiling [38]. Based on the studies conducted so far, microdroplet technology could be used to process DNA for the massively parallel amplification of specific subsets of the human genome for targeted sequencing. More evidence must be obtained before microdroplet PCR can be applied for routine diagnostic purposes.

#### 2.1.2. Non-PCR-Based Strategies

The so-called “sequence capture” approach, unlike PCR, is an excellent way to isolate large or highly dispersed regions from a pool of DNA molecules [39]. Sequence capture is essentially based on hybrid capture reactions for the selective enrichment of targeted genomic regions. Specific capture probes can be synthesized to enrich the regions of interest from the whole genome, thus obtaining a captured, adapted, and barcoded library for NGS applications [9, 40, 41]. In detail, DNA fragments hybridize to the capture probes synthesized on DNA microarray glass slides in array-based hybridization [9], whereas biotinylated DNA or RNA probes are used in liquid-phase hybridization. The nontargeted DNA fragments are washed away and the enriched DNA is recovered and used for high throughput sequencing. Currently, three major commercial products, namely, Nimblegen's SeqCap (array-based and solution-based), Illumina's TruSeq (solution-based), and Agilent's Sure-Select (array-based and solution-based), are used in combination with NGS platforms (e.g., Illumina, Roche 454, and Solid) to achieve efficient target enrichment. In addition to these, the HaloPlex technology from Agilent (solution-based) is a well-known example of an enrichment system featuring a selective circularization-based method that is a further development of the principle of selector probes used in several diagnostic approaches [42, 43].

Although PCR-based enrichment methods have the benefit of even coverage and high specificity, in some cases DNA sequence capture is to be preferred because hybridization is less sensitive to contamination and the procedure is less vulnerable to mismatches. In addition, PCR specificity depends on reaction optimization and primer design: large rearrangements in genomes, for example, may be undetectable. Unlike PCR, hybridization capture requires relatively large amounts of high-quality DNA and target molecules may be lost during library preparation. Therefore, this approach is more suitable for the study of large genomic regions, either contiguous or not contiguous, including the entire exome, as discussed in greater detail under “*Whole-Exome Sequencing*” and “*Targeted DNA Sequence Capture Sequencing*” below.

*Whole-Exome Sequencing*. Whole-exome sequencing (WES) serves to selectively sequence the coding regions of a genome in order to discover rare or common variants associated with a disorder or phenotype [44, 45]. In humans, the exome represents approximately 1% (30 megabases (Mb)) of the genome and it accounts for over 85% of disease-causing mutations [10, 46]. Consequently, WES is an attractive and practical approach for the study of coding variants related to rare Mendelian disorders and of many disease-predisposing single nucleotide polymorphisms throughout the exome [47].

Various target enrichment strategies are used in exome sequencing to pull out the coding regions from the whole genome, namely, array-based technologies, multiplex PCR, selector probe (HaloPlex), solution hybridization (Illumina TruSeq, Agilent SureSelect, and NimbleGen SeqCap EZ), and molecular-inversion probes (MIPs) [47–50]. Each approach has its capture efficiency and the choice of one approach over another depends mainly on the researcher's preference and needs [51, 52].

The last few years has seen an exponential growth in WES studies. This technique has been successfully used to identify the causative variations in such heterogeneous conditions as hearing loss [53–57], monogenic types of diabetes [58, 59], nonsyndromic mental retardation [60], and lysosomal disorders [61]. In addition, WES has become a tool in routine clinical practice [62]. One example is the case reported by Choi et al. in 2009 [63] of a patient supposedly affected by Bartter syndrome, a recessive renal tubular disorder characterized by hypokalemia and hypochloremia, who carried a novel homozygous mutation in the SLC26A3 gene that is known to cause congenital chloride-losing diarrhea. Therefore, the patient underwent a clinical reevaluation that led to a final diagnosis of congenital chloride-losing diarrhea.

An exciting new application of WES is in the prenatal diagnosis of aneuploidies. Fetal DNA can be extracted from the plasma fraction of maternal peripheral blood thereby avoiding an invasive approach [64–69]. A very recent paper published in the New England Journal of Medicine showed that prenatal testing on plasma-cell free DNA had significantly lower false positive rates and higher positive predictive values in detecting trisomies 21 and 18 than standard screening [70].

Yet another field of WES application is in cancer genetic research. Human cancer is characterized by the accumulation of genetic alterations and WES is an optimal tool for multiple gene testing. For example, in the field of the molecular genetics of cancer, WES has been applied in gastric cancer [71, 72], lymphomas [73, 74], breast cancer [75], melanomas [76, 77], and prostate cancer [78, 79]. Recently, WES was used to study circulating tumor cells in metastatic prostate cancer and revealed, with high accuracy, single nucleotide variants that can potentially track tumor evolution, guide therapy, and monitor relapses [80]. Cost-effective targeted WES methods have also resulted in a step forward in the field of personalized chemotherapy [81] and are now being used in the fields of pharmacogenetics and personalized medicine in general to identify inherited genetic variants able to predict individual responses to specific treatment. In fact, Daneshjou et al. [82] sequenced the entire exome of about 100 individuals and identified the genetic factors associated with the response to different doses of warfarin. More recently, using WES, Apellániz-Ruiz et al. [83] identified rare* CYP3A4* variants associated with individual susceptibility to toxicity to paclitaxel, which is a frequently used chemotherapy agent.

To sum up, WES has many strengths: it is less expensive than whole-genome sequencing (WGS) (one exome costs USD 1,000 whereas one genome costs around USD 2,000) [6], it is an efficient strategy with which to identify the genetic basis that underlies rare Mendelian disorders, and it provides a small dataset compared to WGS and is thus easier to interpret. On the other hand, exome sequencing obviously analyzes only about one percent of the entire genome and leaves out noncoding regions, such as untranslated regions (5′UTR and 3′UTR), promoters, and other potentially functional regions. This limitation could be overcome by customizing commercial exome capture kits according to the researcher's scientific needs. Other limitations and challenges related to WES are genetic heterogeneity (where several genes are associated with the same disorder), natural duplicated sequences throughout the genome, and pathogenic mutations located in noncoding genes, have corresponding pseudogenes, contain repetitive or high CG-rich regions, or are within the mitochondrial genome which are not detected. In addition, large deletions/duplications/rearrangements and mosaicism may not be detected by WES.

Finally, hybridization methods like WES may show some biases when analyzing repetitive regions versus PCR-based enrichment strategies and may not be able to efficiently enrich them. This uneven sequence coverage should be carefully evaluated in the diagnostic context since it could result in the loss of clinically relevant sequence variations and, thus, in false-negative results. Recently, Patwardhan et al. demonstrated that “augmented exome sequencing,” which is designed to increase sequence coverage in medically relevant regions and in difficult-to-sequence regions, was more efficient in the clinical setting than other WES strategies [84].

*Targeted DNA Sequence Capture Sequencing*. Target enrichment strategies enable the efficient and rapid querying of specific large genomic regions of interest. Using this approach, one can isolate genomic regions in a library and thereby quantitate both germline and somatic variants. As mentioned in Section 2.1.2, various hybridization-based enrichment methods, either in solution or on array, have been developed [85, 86]. Targeted enrichment-based approaches necessitate the identification of the target regions of interest and appropriately designed probes because repeating elements and high guanine-cytosine content hamper complete target coverage. Repetitive elements and internal duplications that may lead to cross-hybridization are usually removed with repeat-masked methods [87]. Long oligo probes (>50 base pairs (bp)), generally based on information from web-based tools (e.g., UCSC, Ensemble, and RefSeq Database), should be designed so as to increase probe specificity. Enrichment reliability can be evaluated based on several parameters: sensitivity and specificity, that is, the percentage of target bases that are represented by sequence reads and the percentage of sequences that map to the intended targets, respectively, uniformity of enrichment results and reproducibility of sequencing runs. Enrichment is considered good when more than 60% of reads map against the target regions. In addition, high coverage (>30x–40x) improves sequencing accuracy. Many enrichment protocols have been tested to increase target enrichment efficiency [88]. Compared to WGS, targeted resequencing can yield a much higher coverage of genomic regions and reduce the time and cost of the analysis. Currently, Illumina sequencing seems to dominate the sequencing market thanks to its lower cost and the shorter time required to process a large number of samples [51].

In terms of accuracy, Illumina sequencing provides a good data backbone and overcomes the homopolymer errors that occur with other sequencing technologies; consequently, it performs better than other strategies in the coverage of medically relevant DNA sequence variations [89, 90].

In conclusion, given the above and the fact that target enrichment technologies are easy to use, these methodologies lend themselves to the study of the molecular basis of genetic diseases, for both research and diagnostic purposes. Thus far, target enrichment-based strategies have been used to identify nucleotide variants [91, 92] and to validate novel diagnostic tools [93–96] and for drug resistance/sensitivity profiling [97–99]. This method is useful for the study of complex families with different genotype/phenotype correlations and to identify at-risk subjects [100]. Finally, hybridization-based targeted sequencing of single nucleotide polymorphism loci in maternal plasma DNA is a promising noninvasive approach to the prenatal diagnosis of fetal chromosomal anomalies [101]. Targeted enrichment technologies can be used to study not only monogenic and polygenic diseases [102–106], but also mitochondrial diseases [107], somatic mutations in tumors [108], and chromosomal anomalies [109]. Importantly, in all these settings, the analysis can be extended to the noncoding regions. Thus, although WES has some limitations in terms of data analysis, management, and interpretation, target capture enables the study of a more restricted and personalized target genomic region, thereby simplifying data analysis and reducing experimental time and costs.

#### 2.1.3. Whole-Genome Sequencing

In the previous sections, we have reviewed the different analytical presequencing strategies currently available for the enrichment of specific genomic regions of interest (from PCR-based approaches to WES) and able to identify gene sequence variations at DNA level. Although not yet suitable for routine applications due to costs, analytical time required, and the huge amounts of data produced, WGS has some advantages over enrichment strategies and is briefly discussed in this section.

Whole-genome sequencing means the sequencing of the human genome in its totality. Therefore, it allows greater sequencing coverage uniformity and can identify also copy number variations, large insertions/deletions, and gene fusions. In addition, WGS covers all the genomic noncoding regions, including introns, promoters, UTRs, and regulatory elements; thus, it can shed light on the molecular alterations involved in specific diseases [110, 111]. Recently, Belkadi et al. compared the performances of WES and WGS in 6 individuals and found that sequencing quality was better and the detection rate of variant higher with WGS than with WES [112].

Whole-genome sequencing has also been applied in the fields of pharmacogenetics and pharmacogenomics [113]. For example, Mizzi et al. analyzed 482 unrelated individuals and identified several pharmacogene-related variants potentially involved in a given individual's response to treatment [114]. Notably, WGS-based approaches are effective tools for large population-based studies. Nagasaki el al. sequenced about 1,000 Japanese individuals and obtained a large population-specific dataset of DNA variants that is useful for epidemiological evaluations [115].

Finally, WGS has been used also to study the molecular basis of human diseases. A study involving 50 patients with severe intellectual disability showed the potential of this approach for the molecular diagnosis of complex diseases that can be caused by diverse kinds of mutations, including* de novo* mutations and copy number variants [116]. Whole-genome sequencing in a patient affected by familial adenomatous polyposis revealed an* APC* mosaicism, suggesting that WGS could be a powerful tool also in the detection of genetic mosaicism related to disease onset [117]. The WGS of a large cohort of patients with early onset familial Alzheimer disease revealed a disease-specific haplotype and a potential disease-progression modifier [118]. Of course, we expect that the number of studies designed to assess the power of WGS will grow rapidly in the next few years. However, as extensively reviewed elsewhere [110, 111], the applications of WGS for diagnostic purposes are still at an embryonic stage due to its costs and particularly due to problems associated with data analysis, interpretation, and storage. However, it is conceivable that as new bioinformatic pipelines for WGS data handling and interpretation become available and sequencing costs decrease, WGS will become the strategy of choice for studies of DNA sequence alterations.

### 2.2. Genome-Wide DNA Methylation Analysis

DNA methylation is one of the more stable and heritable epigenetic marks and its dysregulation is associated with many human diseases [119]. The human genome is highly methylated; approximately 80% of cytosines in CpG dinucleotides are chemically modified [120]. The assays for identifying methylated CpG dinucleotides in a genome vary in terms of resolution and cost. Although PCR-based DNA methylation approaches have several advantages [121], the ongoing revolution in sequencing technology has opened the door to whole-genome DNA methylation analysis at a single-base-pair resolution. There are three main approaches to whole-genome methylation studies: enzyme digestion [11, 122], affinity enrichment [123–126], and bisulfite sequencing [127–131].

It is difficult to compare these three approaches given their complexity and diversity. The choice of the most suitable method depends on the desired coverage, accuracy, and resolution, as well as on the number of samples and the DNA quality and quantity. In general, enzyme digestion and affinity enrichment-based methods are low-resolution and essentially qualitative. Instead, bisulfite sequencing approaches have a higher resolution and provide quantitative estimates of methylation [132, 133]. Whole-genome bisulfite sequencing can assess about 95% of all CpG sites in the genome; however, high coverage (>500 million paired-end reads to achieve ~30x coverage) and high DNA input (1–1.5 versus 0.2 micrograms (g)) are required [131]; here it should be noted that Illumina's new EpiGnome Methyl-Seq kit requires only 50–100 nanograms (*μ*g) of genomic DNA. Incomplete bisulfite conversion and differential PCR efficiency for methylated versus unmethylated sequences are the main limitations of bisulfite sequencing approaches. Recently, sequence capture enrichment methods have been developed also for DNA methylation assessment at a single base resolution (Nimblegen/Roche SeqCap Epi and Agilent MethylSeq). These approaches are based on the same enrichment methodology used to identify DNA sequence variations for the enrichment of bisulfite-converted DNA. Also in this case, these methods enable genome-wide capture of all the annotated CpG islands and customized enrichment to study pathways of interest [134].

Advances in genome-wide DNA methylation technology have also resulted in new strategies for the timely identification of novel diagnostic and prognostic biomarkers. For example, Huang and colleagues identified functional DNA methylation biomarkers predictive of the clinical outcome of ovarian cancer [135]; Jerónimo and colleagues showed that the glutathione-S-transferase P1 gene is methylated in >90% of prostate cancers [136]. Moreover, 70 genes were found to be significantly hypermethylated in gastric cancer tissue compared with those observed in normal tissue [137]. Ghosh and coworkers used whole-genome DNA methylation profiling to explore a potential association between parity and epigenetic changes in breast tissue from women with early parity and nulliparity. They identified six genes that are hypermethylated in the parous group [138]. Furthermore, in a very recent article, Warton and colleagues described a comprehensive technical analysis of free cell DNA (fcDNA) isolation from healthy subjects and enrichment of methylated sequences followed by NGS [139]. Their findings provide further support that whole-genome analysis of even small amounts of fcDNA can provide high-quality, validated genomic data that strengthen the potential of the usefulness of the methylation signature of fcDNA in clinical applications. The epigenomic data and the discovery of a specific pattern of epigenomic marks associated with specific functional regions have also helped to clarify genotype-phenotype association data.

### 2.3. Metagenomics

Although metagenomics does not include the study of human genes but focuses on the characterization of microbial communities, namely, microbiota, living in specific environments, such as skin or mucosal districts, growing evidence implicates the human microbiome in the development of various diseases. Therefore, it seems appropriate to mention this technique, although for further details we refer readers to more specific reviews on this topic [12, 140–142]. It is now well known that the human microbiome is required for the maintenance of the healthy status [143] and that microbial dysbiosis could play a role in several diseases like diabetes, inflammatory bowel diseases, obesity, and cancer [144–149]. So, the study of microbiome composition (both qualitatively and quantitatively) could clarify diseases pathogenesis and, in turn, pave the way to the development of novel diagnostic, prognostic, and therapeutic targets. This explains the great interest in the field. NGS-based approaches have greatly impacted also metagenomics, thereby providing a comprehensive view of microbial communities.

As we reviewed elsewhere [150], 16S bacterial ribosomal RNA (rRNA) characterization, obtained using NGS-based strategies, is now the technique most widely used to study the microbiome. It is an amplicon-based method that uses bacterial universal primers to amplify the entire microbiome in one PCR reaction [151]. The complexity of microbial communities is resolved after sequencing, specifically assigning each read to a group of bacteria through specific bioinformatics tools. Using this approach, we monitored the gut microbiome of a patient with Crohn disease before and after nutritional therapy and showed that this therapy was effective in restoring gut microbiome dysbiosis [152]. The same strategy has been recently used to characterize the esophageal microbiome in eosinophilic esophagitis [153], the lung microbiome of cystic fibrosis patients [154], and the subgingival microbiota in different periodontal diseases [155], and other studies are appearing almost daily. It is conceivable that future technical advances (especially those related to data analysis tools and to the availability of microbial community databases) will shed light on the functions of the human microbiome and its role in human diseases, and metagenomics could be an integrative means with which to study the molecular basis of human diseases at DNA level.

### 2.4. RNA Sequencing

The key to the molecular basis of human diseases and the genotype-phenotype relationship lies in gene expression and the mechanisms that control it. Consequently, genome-wide expression analyses are now pivotal in genomics and biomedical research. RNA sequencing (RNA-seq) technologies are elucidating the mechanisms that expand the genome's coding capacity and are revolutionizing the concept of gene expression regulation. RNA-seq is gradually replacing microarrays in high throughput gene expression studies because it provides more quantitatively accurate measurements and also absolute transcript abundance data [156, 157]. RNA-seq also detects annotated transcripts as well as novel sequences, splice variants, exon junctions, noncoding RNA [158], single nucleotide polymorphisms [159], and fusion genes [160]. Various RNA-seq techniques are available, and the one to use depends on the RNA species being investigated: (i) total RNA sequencing (total RNA-seq) is a process that removes rRNAs and thus captures a broader range of gene expression changes and reveals novel transcripts in both coding and noncoding RNA species; (ii) coding RNA sequencing (mRNA-seq) provides information about poly-A tailed RNAs; and (iii) small RNAs sequencing (small RNA-seq) is used to discover and analyze novel microRNAs (miRNAs) and other small noncoding RNAs.

Changes in the expression of coding genes are controlled at multiple levels, from transcription to RNA processing and translation. Interestingly, the abundance of a transcript is directly modified by polymorphisms in regulatory elements [161]. An important class of variants, called* expression quantitative trait loci* (eQTL), influences the expression level of the gene in two ways (local or distant) [162, 163]. Genome-wide association studies combined with RNA-seq analysis can reveal the eQTL and can shed light on the mechanism whereby gene variability controls gene expression. The development of techniques based on the integration of these data will help to understand putative causal links between DNA variation and expression.

## 3. NGS Data Analysis and Storage

Data management and analysis pipelines, based on bioinformatics expertise and hardware infrastructures, have been developed to manage the massive sets of data produced by NGS. The analysis of NGS data is commonly based on three main analytic steps [164, 165], which are usually implemented via specific bioinformatic tools: (i) generation of sequences and assignment of base quality scores; (ii) demultiplexing (if necessary), read alignment, and variant calling; and (iii) identification and interpretation of variants according to guidelines [166]. Different scripts and/or pipelines are used in this last step depending on the kind of application, the type of samples sequenced, and the biological question to be addressed. For example, specific tools are available for metagenomics [167–169] and RNA-seq studies [170, 171].

Storage of the huge amount of data generated by NGS is an important issue. The Centers for Disease Control and Prevention (http://www.cdc.gov/clia/Resources/GetRM) requires storage of analytic systems records and data reports for at least 2 years [172]. However, given the rapid growth of knowledge in this field, longer storage could be contemplated for raw data files (e.g., fastq files) so that the primary results can be regenerated and analyzed as more advanced accurate techniques become available to verify the original interpretation. Consequently, sequencing centers should be equipped with powerful dedicated storage equipment: it has been estimated that 3.2 terabytes is required for the backup of 200 exomes [173]. These infrastructures may be beyond some laboratories, especially small laboratories. Publicly accessible clouds represent a possible solution to data storage, although, in a clinical context, data privacy issues should be carefully addressed [174].

## 4. Next-Generation Sequencing in the Clinical Setting and Ethical Disclosures

As reviewed in the previous sections, several NGS-based approaches are available to study the molecular bases of human diseases at DNA (and also at RNA) level and are now routinely used in clinical diagnostics [7, 110]. In essence, single gene analysis (multiplex PCR or long PCR) should be restricted to cases of low genetic heterogeneity; gene panel screening (microdroplet PCR or targeted sequence capture enrichment) should be preferred in case of highly heterogeneous diseases and/or for the differential diagnosis of very similar diseases, while WES and WGS should be considered in case of a very complex/rare phenotype, when* de novo* mutations are suspected or in case of noninformative results after the analysis of a panel of targeted genes [175]. The main issue concerning the clinical use of NGS-based approaches is the huge amount of data produced and its interpretation: the greater the genomic region analyzed, the greater the number of variants of uncertain significance identified. Another concern regards the so-called incidental findings, that is, mutation(s) with a known pathogenicity but not related to the medical condition for which the test was requested. Notably, the issue as to whether or not incidental findings should be communicated to the patient is hotly debated. Of course, a carefully produced patient-informed consent procedure should be part of pretest genetic counseling to prepare the patient for such kinds of results and ask for their concerns regarding the knowledge of the results.

## 5. Development of Next-Generation Sequencing Technology

Next-generation technologies were launched on the market about ten years ago and their history is characterized by a continuous release of novel instruments that usually feature an increased throughput/sequencing run [176]. The sample preparation workflow is usually based on three main steps: library preparation (achieved with different strategies depending on the project, as discussed in Section 2), library amplification, and high throughput sequencing. Each instrument uses specific chemistry, which accounts for some differences in sequencing accuracy and quality. The features of specific NGS platforms have been extensively reviewed and compared elsewhere, Illumina technology being the most widely used procedure to date [89]. Here, we provide an overview of the more recently developed technologies that may undergo further improvements in the next few years and possibly replace currently used platforms.

Pacific Biosciences has developed a NGS platform based on the real-time sequencing of single molecules (SMRT) during polymerization reactions. The DNA polymerase is immobilized on the bottom of microscope chambers: the four phosphate-labelled nucleotides are eluted in the chambers and the sequences are read in real-time since fluorescent specific signals are recorded after incorporation of each nucleotide [177]. This procedure avoids library amplification and consequently reduces the risk of PCR artefacts. The latest version of this instrument has 150,000 chambers each able to sequence 55,000 reads/run with an average length of 20 kb and a maximum throughput of 1 gigabase per chamber in a four-hour run. The absence of clonal amplification, together with the long read length, makes this technology appealing for a variety of applications [178, 179]; however, the error rate is still high [180].

Another promising single molecule sequencing method is the nanopore-based strategy developed by Oxford Nanopore Technologies. It is based on a flow-cell containing hundreds of microwells. Each microwell contains a membrane with a nanopore through which an ionic current flows. When a biological molecule (DNA, RNA, or protein) flows through the pore, each nucleotide/amino acid results in a specific current change that discriminates among them [181]. Oxford Nanopore Technologies has developed three scalable instruments, including the MinION, a USB-device portable sequencer able to generate up to 16,000 reads/run with a maximum length of 60 kb and an average throughput of 90 Mb in an 18-hour run. Also in this case, the error rate is still too high for routine use. Finally, Complete Genomics has launched a fully automated apparatus for large-scale WES and WGS currently available only as a service. The instrument is based on Combinatorial Probe-Anchor Ligation chemistry [182] and is designed to sequence up to 10,000 genomes per year with a 50x coverage.

To overcome the limitations and drawbacks of currently available NGS instruments in terms of productivity, speed, cost, and accuracy, many other sequencers, based on diverse technologies (microfluidics, electron microscopy, nanopore-based strategies, and DNA transistor-based procedures), are currently under development. Therefore, it is conceivable that NGS will become a “routine” procedure in the not too distant future.

## 6. Conclusions

Next-generation sequencing technologies and the various associated procedures, together with the plethora of data they have generated and continue to generate, prompt several basic concepts. Many diseases, if not all, are characterized by genetic changes and thus are related to DNA sequence variants that must be analyzed for their consequences. However, not all these variants shed light on a given disease, since they are not directly pathogenetic or they contribute only slightly to the pathogenesis of the disease. Therefore, to address the complexity of the link between DNA sequence variants and human diseases, high throughput DNA sequencing should be as rapid and inexpensive as possible. Moreover, the sequence and bioinformatic strategies that are continuously emerging, together with the detection of DNA sequence changes, should also take account of DNA modifying processes, such as methylation. In addition, going from DNA to RNA, NGS has enabled researchers to mine the enormous amount of quantitative and qualitative data buried in the myriad of regulatory sequence elements that have been discovered in recent years, namely, miRNA, long noncoding RNA, small circular RNA, nuclear RNA, and nucleolar RNA. Notwithstanding the enormous potential of these methodologies, the procedures and guidelines have yet to be standardized, which probably reflects the continuous innovations that are being made in this field.

Notably, in the clinical context, besides confirming a disease or correlating a gene alteration to a given disease, NGS has the potential to become the frontline analysis for differential diagnosis among clinically confounding diseases. Thus, gene sequence profiling may be able to discriminate one disease from another, therefore enabling timely effective therapy.

The easier and faster production of highly accurate sequence data will certainly give impetus to what is now called “personalized genome analysis.” Besides examining nosographically established diseases that affect many people, personalized genome analysis can help to understand, at single individual level, the minute differences that can affect the health status of each person, which, in turn, can lead to the application of “personalized medicine.”

Another important area of research that has benefitted from NGS technology is the development of target drugs, namely, drugs or compounds that act as bullets able to strike a precise target in a DNA sequence, or at a corresponding protein level, in order to nullify or even to reverse the nucleic sequence from the variant to the wild-type status. This newly emerging technique of gene editing requires very accurate DNA and RNA sequences in order to design the most effective tools with which to revert the altered nucleotide into the wild type. This is a very promising avenue of research, provided the related ethical issues are overcome.

Finally, NGS-based approaches have greatly improved our understanding of the molecular basis of human diseases in a variety of ways that were unthinkable just a few years ago. The challenge now is to resolve the outstanding issues of standardization of procedures, the production and storage of personal data, and other ethical aspects, which we suspect will animate scientific and regulatory debates in the next few years.

## Figures and Tables

**Table 1:** Currently available NGS-based approaches for the study of the molecular basis of human diseases at DNA level.

Enrichment system	DNA input	Analytical time^*∗*^	Sensitivity	Specificity	Max target size	Pros/cons
Long PCR	5 ng/amplicon	4-5 h	High	High	Depends on amplicon length	Rapid analysis of large genes/excellent quality of template DNA required; longer read lengths; greater fidelity and higher yields of Taq DNA polymerase; optimization of primer design and amplification conditions.

Multiplex PCR	5 ng/multiplex	3-4 h	High	High	Depends on amplicon length and multiplexing	Simultaneous analysis of several different targets; significant time and cost reductions/primer design and amplification conditions optimization; analysis of specific genes sequences only.

Microdroplet PCR	1.5 *μ*g	48 h^†^	High	High	Up to 20,000 genomic loci	Analysis of a large number of genes in a single sequencing run; rapid high-quality data production/specific instrument acquisition required; high-quality DNA required; complex data analysis; high costs.

WES	500 ng–2 *μ*g	92 h	High	>60%^§^	50–75 Mb^§^	High coverage in targeted regions; reduced costs for large genome analysis/mutations in regulatory regions will be missed; hard to identify structural variants and copy number variations; high sequencing depth required; complex data analysis.

Targeted capture	500 ng–2 *μ*g	92 h	High	>60%^§^	Up to 50 Mb of custom regions	High resolution; cost reduction by pooling tagged samples; less susceptible to contamination and to mismatches/large amount of high-quality DNA required; influenced by repeating elements and high values of guanine-cytosine content; complex data analysis.

WG bisulfite sequencing	50 ng–1.5 *μ*g	30 h	High	95% of CpGs	WG	High resolution and quantitative data/high DNA and sequencing coverage requirements; influenced by efficiency of bisulfite conversion; high cost per sample.

WES, whole-exome sequencing; WG, whole genome; Mb, megabase.

^*∗*^In hours (h) and without considering sequencing time.

^†^Calculated considering 48 samples amplified/run.

^§^Depending on the design and on the commercial enrichment technology used.

**Table 2:** Principal clinical applications of NGS technologies to the study of the molecular basis of human diseases.

Clinical application	NGS approaches
Variant detection	Long PCR [15, 18, 19] Multiplex PCR [16, 20–30] Microdroplet PCR [31, 32] WES [47–50, 53–59, 67–75] Targeted capture [86–88, 91–96] WGS [110–112, 115–118]

Prenatal diagnosis	WES [60, 64–70] Targeted capture [101]

Circulating tumor cells analysis	WES [80]

Pharmacogenomics	WES [81–83] Targeted capture [97–99] WGS [113, 114]

Gene expression regulation	Microdroplet PCR [38] WG bisulfite sequencing [132–139] RNA-seq [156–162]

WES, whole-exome sequencing; WG, whole genome.
